# Specialized Pro-Resolving Lipid Mediators Distinctly Modulate Silver Nanoparticle-Induced Pulmonary Inflammation in Healthy and Metabolic Syndrome Mouse Models

**DOI:** 10.3390/nano14201642

**Published:** 2024-10-13

**Authors:** Arjun Pitchai, Akshada Shinde, Jenna N. Swihart, Kiley Robison, Jonathan H. Shannahan

**Affiliations:** School of Health Sciences, College of Health and Human Sciences, Purdue University, West Lafayette, IN 47907, USA

**Keywords:** pulmonary inflammation, AgNP, metabolic syndrome, specialized pro-resolving mediator, treatment

## Abstract

Individuals with chronic diseases are more vulnerable to environmental inhalation exposures. Although metabolic syndrome (MetS) is increasingly common and is associated with susceptibility to inhalation exposures such as particulate air pollution, the underlying mechanisms remain unclear. In previous studies, we determined that, compared to a healthy mouse model, a mouse model of MetS exhibited increased pulmonary inflammation 24 h after exposure to AgNPs. This exacerbated response was associated with decreases in pulmonary levels of specific specialized pro-resolving mediators (SPMs). Supplementation with specific SPMs that are known to be dysregulated in MetS may alter particulate-induced inflammatory responses and be useful in treatment strategies. Our current study hypothesized that administration of resolvin E1 (RvE1), protectin D1 (PD1), or maresin (MaR1) following AgNP exposure will differentially regulate inflammatory responses. To examine this hypothesis, healthy and MetS mouse models were exposed to either a vehicle (control) or 50 μg of 20 nm AgNPs via oropharyngeal aspiration. They were then treated 24 h post-exposure with either a vehicle (control) or 400 ng of RvE1, PD1, or MaR1 via oropharyngeal aspiration. Endpoints of pulmonary inflammation and toxicity were evaluated three days following AgNP exposure. MetS mice that were exposed to AgNPs and received PBS treatment exhibited significantly exacerbated pulmonary inflammatory responses compared to healthy mice. In mice exposed to AgNPs and treated with RvE1, neutrophil infiltration was reduced in healthy mice and the exacerbated neutrophil levels were decreased in the MetS model. This decreased neutrophilia was associated with decreases in proinflammatory cytokines’ gene and protein expression. Healthy mice treated with PD1 did not demonstrate alterations in AgNP-induced neutrophil levels compared to mice not receiving treat; however, exacerbated neutrophilia was reduced in the MetS model. These PD1 alterations were associated with decreases in proinflammatory cytokines, as well as elevated interleukin-10 (IL-10). Both mouse models receiving MaR1 treatment demonstrated reductions in AgNP-induced neutrophil influx. MaR1 treatment was associated with decreases in proinflammatory cytokines in both models and increases in the resolution inflammatory cytokine IL-10 in both models, which were enhanced in MetS mice. Inflammatory responses to particulate exposure may be treated using specific SPMs, some of which may benefit susceptible subpopulations.

## 1. Introduction

Increased utilization of engineered nanomaterials in chemical, electronic, and medical industries has raised significant concerns about potential inhalation risks [1,2,3,4,5,6]. Simultaneously, ultrafine particulate matter (PM_0.1_) levels are increasing, resulting in human exposure [7,8]. Exposure to these nano-sized particles is especially concerning due to their small size, which results in large particle surface areas, deposition deep within the lung, and enhanced toxic effects. Inhalation of engineered nanoparticles has been linked to numerous negative biological outcomes, including carcinogenicity, genotoxicity, inflammation, and fibrosis [9,10]. Silver nanoparticles (AgNPs) are one of the most produced nanomaterials and have been examined extensively for toxicity outcomes. Specifically, rats exposed to AgNPs developed pulmonary fibrosis and experienced reduced lung function [11,12]. Their toxic effects are linked to AgNP-induced pulmonary oxidative stress and inflammation [13]. Most evaluations of nanoparticle safety have traditionally focused on healthy models; however, a significant portion of our population have underlying health conditions which may alter their sensitivity to exposure.

Recent evidence has demonstrated that individuals with metabolic diseases such as metabolic syndrome (MetS) are more vulnerable to the adverse health effects of particulate air pollution [14]. Specifically, individuals with MetS are more susceptible to negative health effects such as cardiovascular issues, variability in heartbeat, altered cardiac repolarization, and exacerbated pulmonary inflammation due to the inhalation of nano-sized particulate matter in comparison to healthy individuals [15,16,17]. Our research has demonstrated that exposure to AgNPs enhances acute lung inflammatory responses in a MetS mouse model in comparison to a healthy mouse model [18,19,20]. A component of MetS and many other diseases is the dysregulation of lipids [21,22]. Lipids are potent signaling molecules regulating the immune system and mediating the inflammatory response. Inflammation initiated by exposure are organized processes beginning with elevations in lipid mediators of inflammation (LMI), enabling the acute inflammatory response. This proinflammatory state is eventually counteracted by specialized pro-resolving lipid mediators (SPMs), allowing for a return to homeostasis. If not resolved, chronic inflammation can lead to the progression of various diseases [23,24,25]. Primary SPMs include resolvins, lipoxins (LXs), protectins, maresins, and annexins and their signaling results in inflammatory resolution [26]. Inhalation exposure alters pulmonary lipid mediators involved in inflammatory signaling, likely contributing to pulmonary injury [27,28]. A recent study from our laboratory observed unique decreases in multiple SPM precursors and SPMs in the lungs of MetS mice 24 h after exposure to AgNPs, while no SPM alterations occurred in the healthy mouse model [29]. These SPM deficiencies may inhibit the resolution process, leading to persistent inflammation and toxicity [30,31].

Due to their dysregulation in disease scenarios and following exposure, modulation of SPMs may be an effective strategy to address exposure-induced inflammation, thereby protecting tissues from damage. Preventative therapy with a mixture of SPM precursors was determined to attenuate ozone-induced pulmonary inflammation, indicating that SPMs can reduce pulmonary responses to inhalation exposure [32]. Our recent study demonstrated that treatment of a MetS mouse model with either 14-HDHA or 17-HDHA prior to AgNP exposure significantly reduced the exacerbated pulmonary inflammatory response [29]. In a separate study, MetS and healthy mice were treated with RvD1 24 h after AgNP exposure and treatment mitigated the exacerbated effects observed in the MetS model [19]. Currently, there remains a knowledge gap regarding which other SPM deficiencies, beside RvD1, model could be targeted post-exposure in MetS mouse to alleviate exacerbated inflammatory responses.

In our previous evaluation, multiple other SPMs were observed to be distinctly reduced in the lungs of the MetS model following AgNP exposure [20]. These included resolvin E1 (RvE1), protectin D1 (PD1), and maresin-1 (MaR1), which could be useful in treatments to address the toxicity associated with particulate matter exposure as well as the susceptibility to inhaled exposure presented by some groups. RvE1 is biosynthesized from the ω-3 polyunsaturated fatty acid eicosapentaenoic acid (EPA) during the resolution phase of acute inflammation via leukocyte 5-lipoxygenase biosynthesis [33,34]. RvE1 binds to ChemR23 and the leukotriene B4 (LTB4) receptor BLT1 [35,36], thus signaling decreases in neutrophil migration, the reduction of inflammatory cytokine production, generation of the pro-resolving mediator lipoxin A4 [36], and phagocytosis of apoptotic neutrophils by macrophages [33]. PD1 is an ω-3 fatty acid synthesized from docosahexaenoic acid (DHA), generated in human airways [37] and lungs [38]. PD1 activates signaling responses via the GPR37 (parkin-associated endothelin-like receptor/Pael-R) receptor, resulting in increased macrophage phagocytosis and altered cytokine release [39]. PD1 promotes resolution in the lung and blocks airway hyper-responsiveness [37]. A prior study found that PD1 could assist in resolving inflammation in a mouse model of lipopolysaccharide (LPS)-induced acute lung injury (ALI) [40,41]. MaR1 is a lipid mediator and one of the ω-3 fatty acid derivatives formed from DHA, that has pro-resolving abilities [42]. MaR1’s protective actions are mediated by leucine-rich repeat-containing G protein-coupled receptor 6 (LGR6) signaling. In a peritonitis mouse model, MaR1 administration decreased neutrophil infiltration and enhanced the macrophage phagocytic capacities. Further, in lipopolysaccharide-induced acute lung injury, MaR1 treatment decreased pulmonary edema, neutrophil influx, and the production of proinflammatory mediators [42,43,44,45]. Overall, these SPMs have distinct signaling pathways that facilitate inflammatory resolution and may be beneficial for particulate exposure-induced pulmonary inflammation in susceptible subpopulations.

In the current study, we selected RvE1, PD1, and MaR1 to investigate based on recent findings indicating that levels of these SPMs decreased after AgNP exposure, suggesting their role in the pulmonary inflammatory response. Further, addressing these deficiencies may enable therapeutic interventions. Therefore, we hypothesize that AgNP exposure induces heightened pulmonary inflammation in a MetS mouse model compared to a healthy mouse model, which can be alleviated by treatment with RvE1, PD1, and MaR1 to address deficiencies in resolution mediators. To investigate this hypothesis, healthy and MetS mice were exposed to AgNPs and treated with RvE1, PD1, or MaR1 24 h following exposure. Pulmonary endpoints of inflammation and lipid regulation were evaluated 3 d post-exposure to determine the benefits of these treatments in addressing AgNP-induced toxicity.

## 2. Materials and Methods

### 2.1. AgNP Characterization

Silver nanoparticles (AgNPs) with a diameter of 20 nm coated with citrate and suspended in water at 1 mg/mL were purchased from NanoComposix (San Diego, CA, USA). To confirm the manufacturer’s specifications, AgNPs at a concentration of 25 μg/mL in deionized (DI) water were assessed for hydrodynamic size, polydispersion, and ζ-potential using ZetaSizer (Zeta-Sizer Nano, Malvern). Measurements for AgNP characterization are expressed as mean ± standard deviation (n = 4).

### 2.2. Animal Models, Diet-Induced Metabolic Syndrome, AgNP Exposure, and SPMs Treatment

Six-week-old male C57BL/6 J mice were purchased from the Jackson Laboratory (Bar Harbour, ME, USA) and used to generate healthy and MetS mouse models. Mice were provided with either a healthy diet containing 10% kcal fat and 51.6 mg/kg cholesterol (D12450B, Research Diets Inc., New Brunswick, NJ, USA) or a high-fat western diet containing 60% kcal fat and 279.6 mg/kg cholesterol (D12492) for 14 weeks. At 20 weeks of age, the mice were exposed to either 50 μL of water (exposure control) or 50 μg of 20 nm AgNPs by oropharyngeal aspiration (50 μL of stock AgNPs at 1 mg/mL in water). Twenty-four hours following exposure, the subgroups of mice were treated with 40 μL saline (treatment control) or 400 ng of individual SPMs (resolvin E1, protectin D1, or maresin-1 (Cayman Chemical, Ann Arbor, MI, USA)) in 40 μL saline (100 μg/mL) by oropharyngeal aspiration. This exposure and treatment protocol led to the creation of sixteen groups varying based on disease (healthy or MetS), exposure (control (H_2_O) or AgNP), and treatment (PBS, resolvin E1, protectin D1, or maresin-1). Mice were randomized into either healthy or MetS groups. All exposures, treatments, and necropsies were performed at the same time in the morning. Exposure to the vehicle or AgNPs was performed in an alternating manner over multiple days. Twenty-four hours following exposure, mice received individual treatments (RvE1, PD1, or MaR1). Lipids undergo oxidation upon interactions with the environment, therefore, the lipids were suspended with sterile PBS immediately prior to treatment. To lessen the oxidation each day, only a single treatment was delivered, however, the disease and exposure groups were alternated during the treatment delivery. For the necropsy, healthy and MetS exposed to vehicle or AgNPs were euthanized using a strategy that alternated between the groups. Necropsies were all performed within a two-hour period, beginning and ending at the same time each day and including twenty mice each day. All analysis of endpoints, such as mRNA isolation, real-time RT-PCR, and ELISAs were performed simultaneously for all groups, reducing the variability. Our strategies represent a rigorous experimental approach, accounting for factors such as time by alternating between the groups for all components. All animal procedures were conducted following the National Institute of Health guidelines and approved by the Purdue University Animal Care and Use Committee.

AgNP Exposure Rationale. The amount of AgNPs (50 μg/mouse) was selected based on previous studies performed in our laboratory and others that demonstrated an acute inflammatory response that can be used to study differences due to MetS and/or lipid interventions [19,46,47]. AgNP exposure of 50 μg/mouse has been reported to stimulate inflammation characterized by pulmonary neutrophil cell influx and elevated mRNA expression of inflammatory markers in mouse models [20,47]. Previously, we demonstrated that this AgNP exposure resulted in exacerbated inflammation in a MetS model compared to the healthy model, allowing for the evaluation of lipid treatment strategies [19,29]. Specifically, 50 μg of AgNP/mouse induced reductions in SPMs in the MetS model which were associated with enhanced neutrophilia [20]. To contextualize this exposure in terms of human relevance, an assessment of a manufacturing facility producing AgNPs identified a mass concentration of 288 μg/m^3^ in the injection room [48]. Based on this measurement, 50 μg/mouse would be equivalent to 180 days of human exposure.

Lipid Treatment Rationale. Studies have utilized 400 ng of SPMs to examine inflammatory processes in mouse models [19,49]. Further, our selected treatment doses of 400 ng for RvE1, PD1, and MaR1 match a recent study where 400 ng of resolvin D1 was used to investigate AgNP-induced inflammation in the same mouse models [19]. 

Time Point Rationale. The time periods were chosen to match prior research using oropharyngeal aspiration to expose healthy and MetS mice to the same amount of AgNPs and assess the differential pulmonary inflammation [19]. Specifically, we determined that at 24 h after exposure, there were distinct differences in multiple inflammatory markers and these responses peaked at 3 d following exposure. Individual SPM treatments occurred 24 h after exposure to match our previous investigation of resolvin D1 and allow us to modulate the active inflammatory process prior to it peaking at 3 days.

### 2.3. Model Characterization

Prior to necropsy, body weight was assessed in order to observe changes in body weight associated with diets, exposure, and/or lipid treatment. Cardiac puncture was utilized to collect blood and serum was separated by centrifugation to measure the circulating lipids, including total cholesterol, high-density lipoprotein (HDL), and low-density lipoprotein (LDL) (Bioassay Systems, Hayward, CA, USA) using commercial kits via the manufacturer’s instructions. All samples were assessed in duplicate for assays.

### 2.4. Collection and Assessment of Bronchoalveolar Lavage (BAL) Fluid

Right lung BAL fluid was isolated to determine altered cytology and evaluate markers of inflammation and injury following exposure and lipid treatment procedures. On necropsy, the left bronchiole was tied off and the trachea was cannulated with a 20-gauge sterile syringe catheter. BAL fluid from the right lung was collected by carefully washing it four times with individual volumes of cold phosphate buffer saline (PBS) determined by body weight (17.5 mL/kg body weight). The initial lavage was separately collected, processed (centrifuging at 1800 rpm for 10 min at 4 °C), and stored at −80 °C. The initial lavage samples represent the protein-rich aliquot and are typically used for assessment of pulmonary total protein and inflammatory cytokines/chemokines. Total protein was measured using the BCA assay (Thermo Scientific, Hercules, CA, USA). Inflammatory cytokines and chemokines were measured within the BAL fluid with ELISA (described below). To determine the total cell counts, BAL fluid pellets from all four washes were resuspended in PBS, mixed, and counted using a Countess™ 3 FL Automated Cell Counter (Invitrogen, Carlsbad, CA, USA). Equivalent numbers of cells were then attached to microscope slides using a Cytospin IV (Shandon Scientific Ltd., Cheshire, UK). A three-step hematology stain (Fisher Scientific, Newington, NH, USA) was then applied to the microscope slides prior to observation under bright-field microscopy. Cell types were identified and counted based on morphology. For each slide, a minimum of 300 cells were counted and the percentages for each cell type were calculated based on the total cell counts for each sample. Slides were counted blindly and the counts were verified by a separate individual. These assessments are standard and have been used to investigate acute lung inflammation and damage to the alveolar capillary barrier in our lab and by other groups [19,20,50].

### 2.5. mRNA Expression Analysis

Using real-time PCR (rtPCR), the genes implicated in proinflammatory responses and resolution pathways were evaluated from the isolated left lung tissues. In summary, 10 mg of lung tissue was homogenized for 30 s at a speed of 5 m/s using 2 mL vials containing 1.4 mm ceramic (zirconium oxide) beads (CK 14 soft tissue homogenizer Precellys, Bertin Technologies, Rockville, MD, USA) and Trizol (500 μL) (Invitrogen, Carlsbad, CA, USA). Following the manufacturer’s instructions, Direct-zol RNA MiniPrep kits (Zymo Research, Irvine, CA, USA) were used to extract the total RNA from the homogenate. RNA concentrations and quality were evaluated by a Nanodrop (Thermo Scientific, Hercules, CA, USA). Following the manufacturer’s instructions, an aliquot of 1 μg of RNA was reverse transcribed into cDNA using an iScriptTM cDNA Synthesis Kit (Bio-Rad, Hercules, CA, USA). To assess the changes in gene expression, inventoried primers from Qiagen (Hidden, Germany) were used for quantitative real-time rt-PCR analysis. Inflammatory markers evaluated included *chemokine* (*C-C motif*) *ligand 2* (*CCL-2*), *interleukin-6* (*IL-6*), *chemokine* (*C-X-C motif*) *ligand 1* (*CXCL-1*), *chemokine* (*C-X-C motif*) *ligand 2* (*CXCL-2*), *tumor necrosis factor-α* (*TNF-α*), and *interleukin-10* (*IL-10*). The lipid metabolism markers assessed for altered mRNA expression included *arachidonate 5-lipoxygenase* (*ALOX-5*), *arachidonate 15-lipoxygenase* (*ALOX-15*), *phospholipase A2* (*iPLA-2*), *cyclooxygenase 2* (*COX-2*) and *epoxide hydrolase 2* (*Ephx-2*). Lastly, lipid receptor gene expression was assessed by measuring the expression of *chemerin receptor 23* (*ChemR-23*) for RvE1, *G protein-coupled receptor 37* (*GPR-37*) for protectin D1, and *leucine-rich repeat containing G protein-coupled receptor 6* (*LGR-6*) for maresin-1. *Glyceraldehyde 3-phosphate dehydrogenase* (*GAPDH*) was used as the internal control for all evaluated mRNA. Each sample was assessed in duplicate for each gene examined. Fold changes were calculated by comparing all individual sample values to the average of the healthy mouse control (the healthy mouse group was exposed to water and treated with saline).

### 2.6. Assessment of BAL Fluid Chemokines and Cytokines

Inflammatory regulating cytokines and chemokines levels, including chemokine (C-X-C motif) ligand 2 (CXCL-2), interleukin-6 (IL-6), and interleukin-10 (IL-10), were quantified from the collected BAL fluid using Mouse DuoSet ELISA kits (R&D Systems, Minneapolis, MN, USA) on the manufacturer’s protocols.

### 2.7. Statistical Analysis

The results are expressed as mean values ± standard error of means (S.E.M.) with eight animals/control groups and twelve animals/exposure groups. The groups were purposefully unequal due to the limited variability expected in the control groups and higher variability expected in the other groups due to the exposure and treatment procedures. All samples were assessed in duplicate for each assay and values were averaged for all endpoints. For the statistical analysis, a three-way analysis of variance (ANOVA) was used to determine the statistical differences between groups with disease (healthy or MetS), exposure (control or AgNP exposure), and treatment (control, RvE1, protectin D1, or maresin-1) as the three factors. The Bonferroni test was utilized for the multi-comparison analysis. All statistical examinations were performed using GraphPad Prism 10.2.3 software (Graph Pad, San Diego, CA, USA) and *p* < 0.05 was considered to be statistically significant.

## 3. Results

### 3.1. Silver Nanoparticle (AgNP) Characterization

To confirm the manufacturer’s product descriptions, AgNPs coated with citrate were characterized at a concentration of 25 μg/mL in deionized water. Characterization determined that AgNPs had a hydrodynamic size of 22.36 ± 0.03 nm, a polydispersity index of 0.21 ± 0.03, and a ζ-potential of −37.73 ± 4.07 mV. These features of AgNPs are presented as mean ± standard deviation (n = 4) and are consistent with both the manufacturer’s specifications and studies previously evaluating AgNP-induced toxicity [51].

### 3.2. Mouse Model Characterization

**Figure 1:**
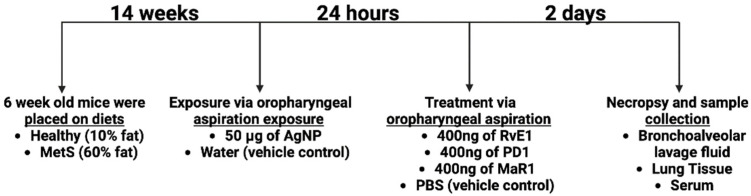
Experiment design timeline. Mice were fed a healthy or high-fat western diet for 14 weeks and exposed to either water (control) or AgNPs (50 µg) via oropharyngeal aspiration; 24 h post-exposure, mice were treated with saline (control) or 400 ng of a lipid resolution mediator (RvE1, PD1, or MaR1) via oropharyngeal aspiration. Endpoints associated with inflammation and lipid metabolism were examined at 2 days following treatment.

**Figure 2:**
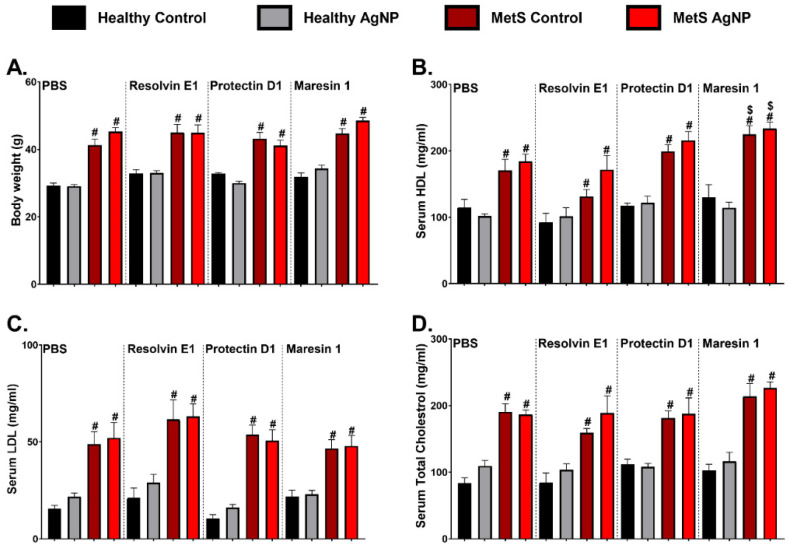
Characterization of (**A**) body weight and serum levels of (**B**) high-density lipoprotein, (**C**) low-density lipoprotein, and (**D**) total cholesterol in healthy and MetS mouse models following 14 weeks of either a healthy or high-fat western diet (HFW diet) and 3 days after oropharyngeal aspiration exposure to pharmaceutical grade sterile water (vehicle) or 50 μg of AgNPs. Subsets of mice were treated with sterile saline (vehicle) or 400 ng of individual SPMs (RvE1, PD1, or MaR1) 24 h post-exposure. Values are expressed as mean ± S.E.M. # disease model, and $ treatment (*p* < 0.05).

Healthy and MetS mice were divided into control (water) and AgNP exposure groups. Twenty-four hours after exposure, the mice received either a vehicle (PBS) or an SPM treatment (resolvin E1 (RvE1), protectin D1 (PD1), or maresin-1 (MaR1). All samples were collected three days following AgNP exposure for assessment of toxicity and inflammatory endpoints (Figure 1). To characterize the healthy and MetS mouse models as well as the potential effects of exposure and treatment, markers of MetS including body weight (BW), serum total cholesterol (TC), high-density lipoprotein (HDL), and low-density lipoprotein (LDL) were measured. The MetS model demonstrated greater body weight compared to the healthy model (Figure 2A). Serum, HDL, LDL and TC were elevated in the MetS mouse model compared to the healthy model (Figure 2B–D). Overall, these results are similar to our previous studies and others utilizing a diet-induced mouse model of MetS [17,19,20,29]. Exposure to AgNPs and/or treatment with RvE1 and PD1 did not change body weight or serum levels of TC, HDL, and LDL in the healthy or MetS mouse models (Figure 2A,C,D). Although MetS mice receiving MaR1 treatment did not demonstrate alterations in body weight, serum levels of LDL or TC compared to MetS mice receiving PBS did demonstrate elevated serum HDL (Figure 2B).

### 3.3. BAL Fluid Markers of Pulmonary Inflammation

**Figure 3:**
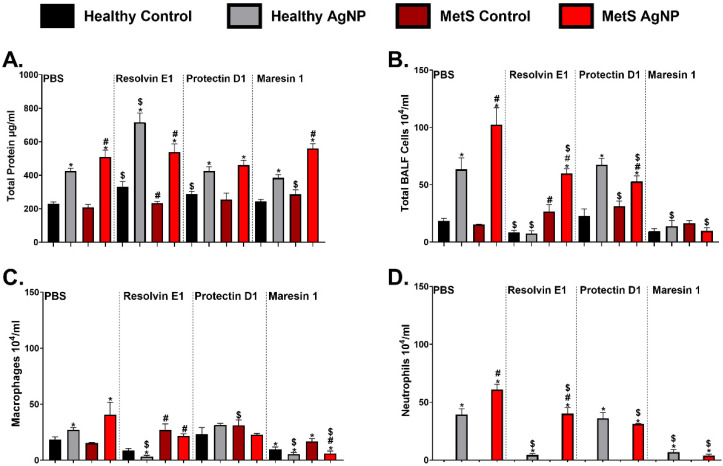
AgNP exposure and modulation by distinct SPM treatment on BALF: (**A**) total protein, (**B**) total cell counts, (**C**) macrophage counts, and (**D**) neutrophil counts from healthy and MetS mice; 24 h following oropharyngeal aspiration of pharmaceutical grade sterile water (control) or 50 μg of AgNPs in sterile water, mice were treated via oropharyngeal aspiration with 400 ng of individual SPMs (RvE1, PD1, or MaR1) or sterile saline (vehicle). Endpoints were evaluated at 3 days post-AgNP exposure. Values are expressed as mean ± S.E.M. * AgNP exposure, # disease model, and $ treatment (*p* < 0.05).

Bronchoalveolar lavage (BAL) fluid was assessed to determine alterations in lung damage and pulmonary inflammatory responses due to AgNP exposure and SPM treatments. Increased pulmonary permeability is indicated by elevated BAL fluid total protein concentration, while alterations in BAL fluid cellular content are indicative of inflammation. Total protein levels, total cell counts, macrophage counts, and neutrophil counts within the BAL fluid were not altered between healthy and MetS controls receiving the PBS treatment (Figure 3). AgNP exposure elevated the total protein levels in BAL fluid in both mouse models receiving PBS treatment (Figure 3A). The MetS mouse model demonstrated enhanced AgNP-induced increases in total protein levels compared to the healthy model. Healthy mice exposed to AgNPs and receiving RvE1 treatment demonstrated increased BAL fluid total protein levels compared to the healthy mice exposed to AgNPs receiving PBS treatment (Figure 3A). Healthy controls receiving PD1 treatment demonstrated elevated total protein levels compared to the healthy controls receiving PBS treatment. (Figure 3A). BALF protein was increased in the MetS controls treated with MaR1 compared to the MetS control treated with PBS (Figure 3A).

AgNP exposure elevated the total cell influx in the BAL fluid in both mouse models treated with PBS at 3 days post-exposure (Figure 3B). The elevation in total cell counts associated with AgNP exposure was exacerbated in the MetS mouse model treated with PBS compared to healthy model (Figure 3B). Healthy control and AgNP-exposed mice receiving RvE1 showed reduced total cell counts in the BAL fluid compared to healthy mice treated with PBS. In the exposed MetS mice, treatment with RvE1 reduced the total cell counts compared to exposed MetS mice receiving PBS. AgNP-exposed MetS models showed a reduction in total cell counts due to PD1 treatment compared to AgNP-exposed MetS mice treated with PBS. Total BAL fluid cell counts were reduced in both models exposed to AgNPs and receiving MaR1 compared to the groups exposed to AgNPs and treated with PBS (Figure 3B). AgNP exposure increased macrophage counts in the BAL fluid of both models receiving PBS treatment (Figure 3C). Macrophage counts were elevated in the MetS controls receiving PD1 and MaR1 treatments compared to the controls receiving PBS. However, macrophage counts were reduced in the healthy exposed mouse models treated with RvE1 and MaR1 compared to the healthy exposed model receiving PBS. (Figure 3C). AgNP exposure increased neutrophil counts in the BAL fluid of healthy mice treated with PBS, which were exacerbated in exposed MetS mice (Figure 3D). Neutrophil counts in the BAL fluid decreased in both exposed models treated with RvE1 and MaR1 compared to mice receiving the PBS treatment (Figure 3D). AgNP-exposed MetS mice treated with PD1 demonstrated a reduction in neutrophils compared to the exposed MetS mice receiving PBS (Figure 3D).

### 3.4. Pulmonary Gene Expression Analysis of Inflammatory Markers

**Figure 4:**
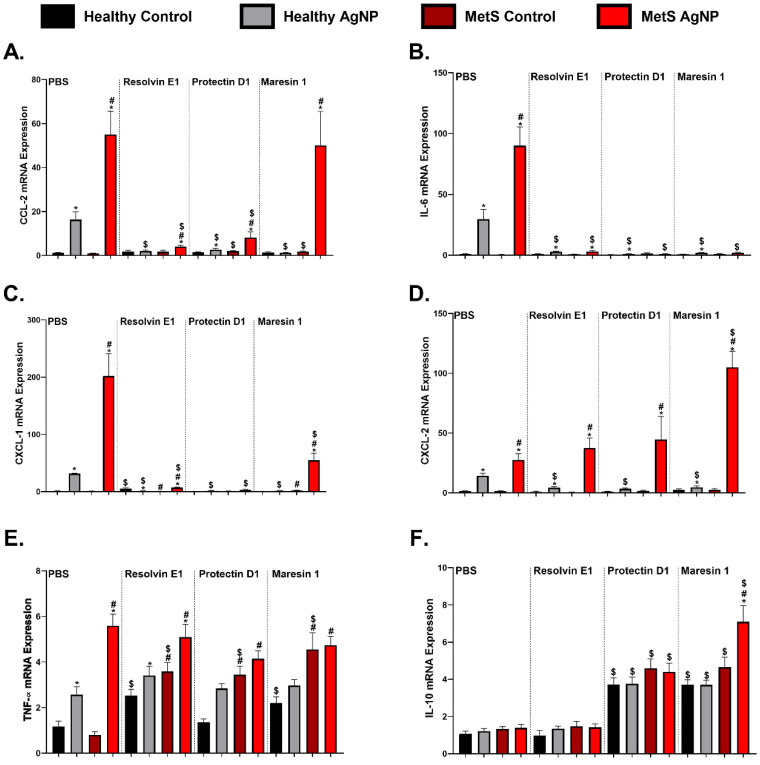
AgNP exposure and modulation by distinct SPM treatment on the pulmonary gene expression of inflammatory factors including (**A**) (C-C motif) ligand 2 (CCL2), (**B**) interleukin-6 (IL-6), (**C**) chemokine (C-X-C motif) ligand 1 (CXCL1), (**D**) chemokine (C-X-C motif) ligand 2 (CXCL2), (**E**) tumor necrosis factor-α (TNF-α), and (**F**) interleukin-10 (IL-10) from healthy and MetS mice; 24 h following oropharyngeal aspiration of pharmaceutical grade sterile water (control) or 50 μg of AgNPs in sterile water, mice were treated via oropharyngeal aspiration with 400 ng of individual SPMs (RvE1, PD1, or MaR1) or sterile saline (vehicle). Endpoints were evaluated at 3 days post-AgNP exposure. Values are expressed as mean ± S.E.M. * AgNP exposure, # disease model, and $ treatment (*p* < 0.05).

Alterations in lung tissue gene expression were assessed to evaluate AgNP-induced inflammation and modulation due to SPMs treatment. Gene expression of *chemokine* (*C-C motif*) *ligand 2* (*CCL-2*), *interleukin-6* (*IL-6*), *chemokine* (*C-X-C motif*) *ligand 1* (*CXCL-1*), *chemokine* (*C-X-C motif*) *ligand 2* (*CXCL-2*), and *tumor necrosis factor-α* (*TNF-α*) were not determined to be different between healthy and MetS models at baseline (unexposed mice receiving PBS treatment) (Figure 4). AgNP exposure elevated proinflammatory cytokine (*CCL-2*, *IL-6*, *CXCL-1*, *CXCL-2, and TNF- α*) mRNA levels in both mouse models treated with PBS, with a more exacerbated response in the exposed MetS mice compared to the exposed healthy mice (Figure 4). *CCL-2* levels decreased in both exposed models when treated with RvE1 and PD1 compared to the PBS-treated exposure groups (Figure 4A). AgNP-exposed healthy mice with MaR1 treatment had a decreased *CCL2* mRNA level compared to the PBS-treated healthy control mice. MetS control mice treated with PD1 and MaR1 demonstrated reduced *CCL2* levels compared to the MetS control receiving PBS (Figure 4A). Pulmonary *IL-6* mRNA levels decreased in both AgNP-exposed models when treated with RvE1, PD1, and MaR1 compared to the exposed PBS-treated mice (Figure 4B). *CXCL-1* mRNA expression was decreased in both exposed models by RvE1, PD1, and MaR1 treatment compared to the treated mice model. (Figure 4C). In the exposed healthy mice, *CXCL-2* levels decreased with RvE1, PD1, and MaR1 treatment compared to the exposed healthy mice receiving PBS. However, in exposed MetS mice, *CXCL-2* levels remained elevated following MaR1 treatment compared to the exposed MetS mice receiving PBS (Figure 4D). *TNF-α* levels were elevated in both control models treated with RvE1 and MaR1 compared to the PBS-treated control models. MetS controls treated with PD1 demonstrated increased *TNF-α* levels compared to the MetS control receiving PBS (Figure 4E).

Gene expression levels related to mediators facilitating resolution were assessed in lung tissue samples to compare the effects of disease and SPM treatment on the AgNP-induced inflammatory response. Gene expression of *interleukin 10* (*IL-10*) did not differ between the healthy and MetS mice in the PBS- and RvE1-treated groups (Figure 4F). Increased levels of *IL-10* were observed in both the exposed and non-exposed models treated with PD1 and MaR1 compared to the PBS-treated mice. In particularl, *IL-10* mRNA was significantly elevated in the exposed MetS mice treated with MaR1 compared to all other SPM-treated mice (Figure 4F).

### 3.5. Pulmonary Gene Expression Analysis of Lipid Metabolism

**Figure 5:**
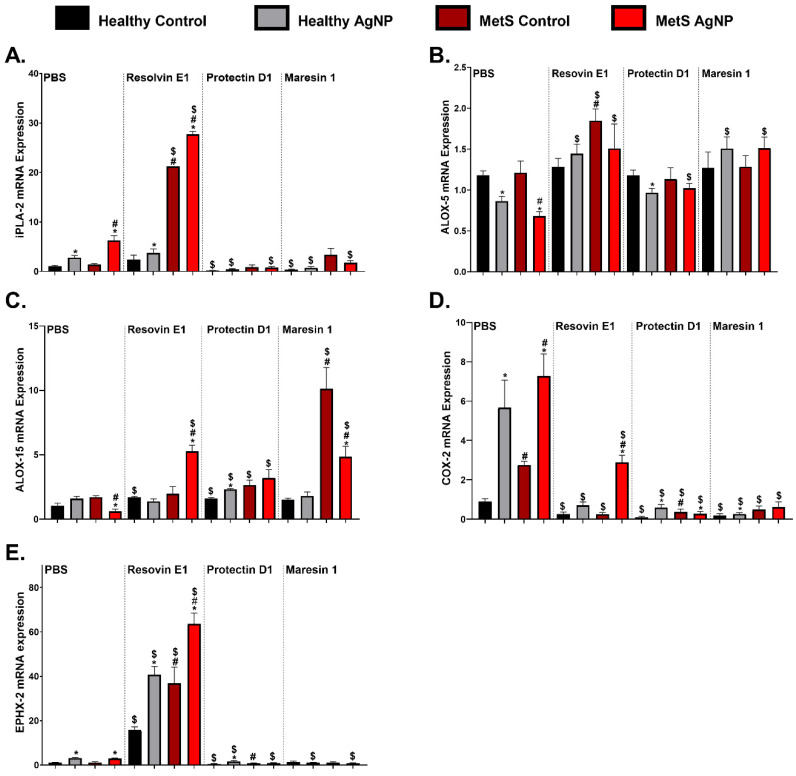
AgNP exposure and modulation by distinct SPM treatment on pulmonary lipid metabolism gene expression, including (**A**) *phospholipase A2* (*iPLA2*), (**B**) *arachidonate 5-lipoxygenase* (*ALOX-5*), (**C**) *arachidonate 15-lipoxygenase* (*ALOX-15*), (**D**) *cyclooxygenase 2* (*COX 2*), and (**E**) *epoxide hydrolase 2* (*Ephx2*) from healthy and MetS mice; 24 h following oropharyngeal aspiration of pharmaceutical grade sterile water (control) or 50 μg of AgNPs in sterile water, mice were treated via oropharyngeal aspiration with 400 ng of individual SPMs or sterile saline (vehicle). Endpoints were evaluated at 3 days post-AgNP exposure. Values are expressed as mean ± S.E.M. * AgNP exposure, # disease model, and $ treatment (*p* < 0.05).

Lipid metabolism gene expression levels in lung tissue samples were assessed to identify differences resulting from MetS and SPM treatment, which may regulate AgNP-induced inflammation. Gene expression of *phospholipase A2* (*iPLA-2*), *arachidonate 5-lipoxygenase* (*ALOX-5*), *arachidonate 15-lipoxygenase* (*ALOX-15*), and *epoxide hydrolase 2* (*Ephx-2*) were not different between healthy and MetS PBS-treated control mice. *Cyclooxygenase 2* (*COX- 2*) was elevated in the MetS control mice receiving PBS compared to the healthy control mice receiving PBS (Figure 5). *iPLA-2* was elevated in both AgNP-exposed models receiving PBS and exacerbated in MetS mice. MetS models receiving RvE1 demonstrated an increased level of *iPLA-2* compared to the PBS-treated MetS group. PD1 and MaR1 treatment decreased the *iPLA2* expression in healthy and exposed MetS models compared to the PBS-treated models. (Figure 5A). AgNP exposure decreased the gene expression of *ALOX-5* in both models and exacerbated it inMetS mice. Exposed healthy and MetS mice treated with RvE1 and MaR1 demonstrated increased *ALOX-5* compared to PBS-treated exposed healthy and MetS mice (Figure 5B). MetS control mice treated with RvE1 demonstrated increased *ALOX-5* levels compared to the MetS controls receiving PBS. *ALOX-5* was increased in exposed MetS mice treated with PD1 compared to the exposed PBS-treated MetS mice (Figure 5B). AgNP exposure decreased the gene expression of *ALOX-15* expression in exposed MetS mice receiving PBS (Figure 5C). Healthy controls receiving RvE1 and PD1 treatment demonstrated elevated expression of *ALOX-15* compared to the PBS-treated healthy control group. *ALOX-15* was elevated in exposed MetS mice receiving RvE1, PD1, and MaR1 compared to the exposed MetS receiving PBS-treatment. *ALOX-15* mRNA expression was elevated in exposed healthy and MetS control mice receiving PD1 treatment compared to the exposed PBS-treated healthy and MetS control groups (Figure 5C). AgNP exposure elevated the mRNA expression of *COX-2* in both exposed mouse models which was exacerbated in MetS model (Figure 5D). *COX-2* expression was decreased in both models, and all groups receiving RvE1, PD1, and MaR1 treatment compared to the PBS treatment groups (Figure 5D). *EPHX-2* was increased in both exposed models treated with PBS. Both healthy and MetS models receiving RvE1 treatment demonstrated elevated *EPHX-2* compared to the PBS-treated models. Exposed healthy and MetS models treated with PD1 and MaR1 demonstrated reduced *EPHX-2* compared to the exposed healthy and MetS models receiving PBS. Healthy controls receiving PD1 had decreased *EPHX-2* expression compared to the PBS-treated healthy control (Figure 5E).

### 3.6. Pulmonary Gene Expression Analysis of Receptors

**Figure 6:**
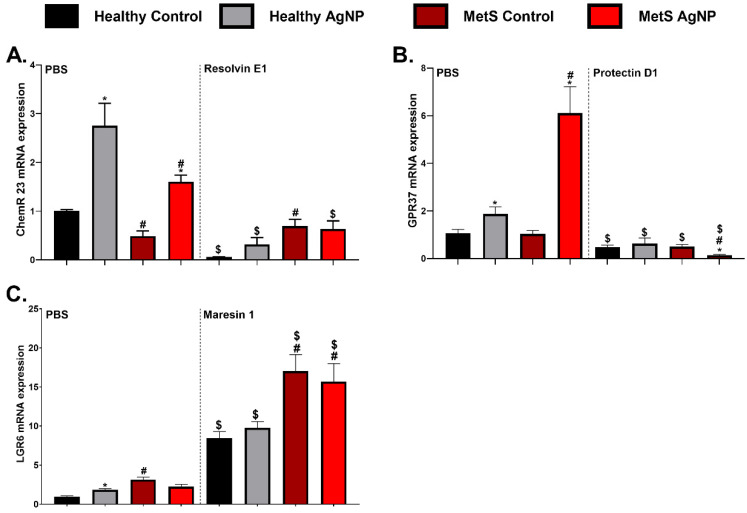
AgNP exposure and modulation by distinct SPM treatment on pulmonary lipid receptor gene expression including (**A**) the RvE1 receptor, *chemerin receptor 23* (*ChemR23*), (**B**) the PD1 receptor, *G protein-coupled receptor 37* (*GPR37*), and (**C**) the MaR1 receptor, *leucine-rich repeat containing G protein-coupled receptor 6* (*LGR6*) from healthy and MetS mice; 24 h following oropharyngeal aspiration of pharmaceutical grade sterile water (control) or 50 μg of AgNPs in sterile water, mice were treated via oropharyngeal aspiration with 400 ng of individual SPMs or sterile saline (vehicle). Endpoints were evaluated at 3 days post-AgNP exposures. Values are expressed as mean ± S.E.M. * AgNP exposure, # disease model, and $ treatment (*p* < 0.05).

Gene expression levels of receptors involved in SPM signaling were assessed to identify differences caused by disease, exposure, and SPM treatment. These receptors are associated with specific SPMs; therefore, specific receptors were assessed for gene expression alterations. These assessments of SPM receptors consisted of the *chemerin receptor 23* (*ChemR-23*) for RvE1, the *G protein-coupled receptor 37* (*GPR-37*) for PD1, and the *leucine-rich repeat containing G protein-coupled receptor 6* (*LGR-6*) for MaR 1. *ChemR-23* was reduced in the MetS model receiving PBS treatment compared to the healthy model receiving PBS (Figure 6A). Exposure to AgNPs increased the *ChemR23* levels in both models receiving PBS compared to the PBS-treated control group. Both healthy models and exposed MetS mice receiving RvE1 treatment demonstrated decreased expression of *ChemR-23* compared to the corresponding PBS-treated groups (Figure 6A). AgNP exposure elevated the *GPR-37* levels in both exposed models receiving PBS and was exacerbated in MetS mice. Decreased *GPR-37* levels were observed in the healthy and MetS mice and in all the groups compared to the PBS-treated healthy and MetS mice models (Figure 6B). *LGR-6* expression increased in the MetS control and the exposed healthy models compared to the healthy control. Increased levels of *LGR-6* were observed in both models and in all the groups compared to the PBS-treated groups (Figure 6C).

### 3.7. Inflammatory Chemokines/Cytokines

**Figure 7:**
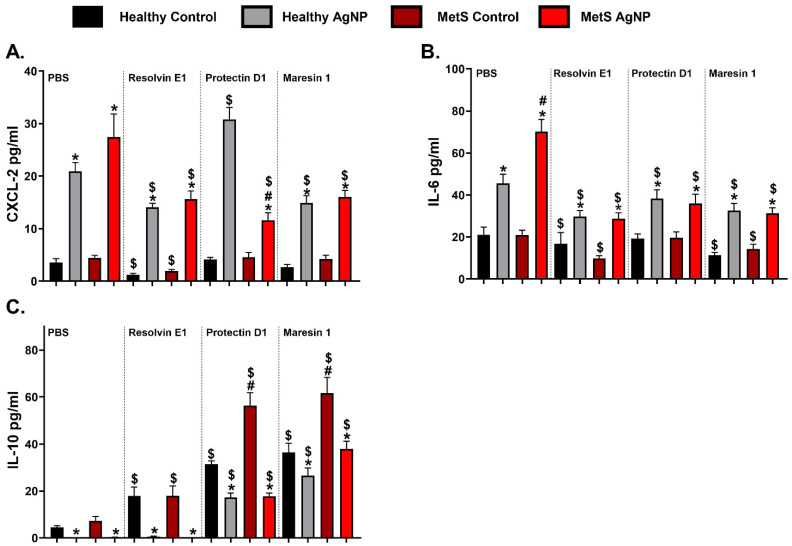
AgNP exposure and modulation by distinct SPM treatment on BALF inflammatory cytokine and chemokine levels including (**A**) chemokine (C-X-C motif) ligand 2 (CXCL2), (**B**) interleukin-6 (IL-6), and (**C**) interleukin-10 (IL-10) from healthy and MetS mice; 24 h following oropharyngeal aspiration of pharmaceutical grade sterile water (control) or 50 μg of AgNPs in sterile water, mice were treated via oropharyngeal aspiration with 400 ng of individual SPMs or sterile saline (vehicle). Endpoints were evaluated at 3 days post-AgNP exposure. Values are expressed as mean ± S.E.M. * AgNP exposure, # disease model, and $ treatment (*p* < 0.05).

To further evaluate the impact of disease and SPM treatment on AgNP-induced pulmonary inflammation, levels of chemokine and cytokine proteins were measured in the collected BAL fluid samples. Concentration levels of chemokine (C-X-C motif) ligand 2 (CXCL-2), interleukin-6 (IL-6), and interleukin-10 (IL-10) chemokine were not changed between models at baseline in the PBS treatment control. AgNP exposure elevated the concentration levels of CXCL-2 in both exposed models receiving PBS treatment, and this induction was exacerbated in MetS mice (Figure 7A). Both control models treated with RvE1 showed a reduced amount of CXCL-2 compared to both controls receiving PBS. Both AgNP-exposed models receiving RvE1 and MaR1 treatment demonstrated reduced CXCL-2 protein levels compared to the exposed PBS-treated groups; however, PD1 treatment only reduced the CXCL-2 level in the exposed MetS model (Figure 7A). AgNP exposure increased the IL-6 levels in both mouse models receiving PBS, which was exacerbated in the MetS model. All healthy and MetS groups receiving RvE1 and MaR1 treatment indicated reduced IL-6 compared to the PBS-treated groups. IL-6 cytokines were decreased in both the exposed mice models receiving PD1 compared to the exposed PBS-treated group (Figure 7B). AgNP exposure decreased the IL-10 in both models of PBS-treated mice (Figure 7C). Both models of control group treatments with RvE1 demonstrated increased levels of IL-10 compared to the control groups treated with PBS. Both models in all the groups receiving PD1 and MaR1 treatment showed increased concentration of IL-10 compared to the group that received PBS treatment. In particular, MetS mice models receiving MaR1 treatment demonstrated elevated IL-10 levels compared to the groups that received PBS and other SPM treatment (Figure 7C).

## 4. Discussion

Individuals with MetS experience heightened inflammation after inhaling particulate matter. Lipids are dysregulated in MetS mice and are involved in the initiation and resolution of inflammatory processes. In our current study, we hypothesized that SPMs (RvE1, PD1, and MaR1) that were previously determined to be dysregulated in the MetS mouse model might be beneficial as treatments to address exacerbated inflammatory responses following AgNP exposure [19]. To test this hypothesis, both healthy and MetS mice were exposed to water (control) or AgNPs to induce an inflammatory response. After 24 h, mice were treated with saline (treatment control) or individual SPMs (RvE1, PD1, MaR1), and pulmonary endpoints related to inflammation and lipid metabolism were evaluated. AgNP exposure resulted in lung inflammation in both mouse models, which was elevated due to MetS, aligning with our previous research. Overall, treatment with RvE1 and MaR1 mitigated the AgNP-induced neutrophila in both models, while PD1 treatment specifically reduced the heightened neutrophila observed in the MetS mice. These data indicate that specific lipid treatments may differentially address AgNP-induced inflammation presented by healthy and MetS mouse models.

AgNPs are one of the nanomaterials most evaluated for toxicity due to concerns regarding their widespread usage [52]. Inhalation is the main route of AgNP exposure, resulting in pulmonary toxicity and inflammation. In particular, a single exposure to AgNPs at the same concentration used in our current study caused inflammation characterized by pulmonary neutrophilic influx, induction of inflammatory cytokines and chemokines, and oxidative stress persisting for 21 days in healthy rodent models [53,54]. There exists a significant knowledge gap concerning the pulmonary toxicity caused by nanoparticle inhalation in common disease conditions. Recently, we demonstrated that AgNP exposure enhances acute inflammatory markers in a MetS mouse model, correlating with reduced levels of pulmonary SPMs at 24 h post-exposure [19,29]. Post-exposure treatment with SPM, RvD1, or pre-exposure treatment with unique SPM precursors (14-HDHA or 17-HDHA) reduced the exacerbation of inflammatory markers in MetS mice following AgNP exposure [19,29]. These findings suggest that dysregulation of SPMs contributed to the increased sensitivity found in a MetS mouse model following AgNP exposure. Further, these dysregulated SPMs could be targeted for therapeutic benefit to lessen the progression of diseases associated with chronic inflammation.

The SPM RvE1 is a potent pro-resolving mediator derived from ω-3 EPA metabolism. Previously, cells treated with EPA and exposed to particulate matter demonstrated reduced pulmonary endothelial dysfunction and inflammation [55,56]. Additionally, treatment with EPA alleviates the oxidative stress and lung inflammation induced by subchronic cigarette smoke exposure in mouse models [57]. These studies suggest that EPA therapies increase EPA-derived SPMs and can reduce lung inflammation following exposures. Treatment with RvE1 has also been demonstrated to reduce lung neutrophil accumulation, improve bacterial clearance, and decrease proinflammatory chemokines and cytokines in the lung following exposure to hydrochloric acid and *Escherichia coli* in mice [33,58]. Our current study supports these previous findings as RvE1 treatment reduced AgNP-induced pulmonary neutrophilia completely in the healthy model, while reducing neutrophilia in the MetS model to levels observed in the healthy mice that did not receive treatment. Treatment with RvE1 inhibited AgNP-induced increases in *CCL-2*, *IL-6*, and *CXCL-1* in both mice models. Additionally, RvE1 treatment reduced *CXCL-2* in AgNP-exposed healthy mice models, but did not alter this response in the MetS model. This continued induction of *CXCL-2* may contribute to the presence of neutrophils observed following RvE1 treatment in the AgNP-exposed MetS model. Previously, CXCL2 levels in serum and mRNA in white adipose tissue were greater in obese individuals than in lean individuals, and immunohistological assessments revealed greater activation of neutrophils [59]. CXCL2 is more potent than CXCL2 in triggering neutrophil movement both in vitro [60] and in subcutaneous air pouches [61], indicating the crucial role of C-X-C motif chemokine receptor 2 (Cxcr2) activity in neutrophilic recruitment [62]. RvE1 has also demonstrated an ability to reduce inflammatory responses to microbial invasion in the lower respiratory tract and promote the resolution of aspiration pneumonia in mouse lungs [58]. These responses were determined to occur independently of the anti-inflammatory cytokine IL-10 [58]. Our results support this previous finding, as RvE1 treatment did not alter *IL-10* mRNA expression at baseline compared to the healthy control mice in either model.

RvE1 is generated via a coordinated metabolism process involving multiple enzymes. The role of phospholipase A2 (PLA-2) is critical in the production of RvE1 and other mediators as it cleaves and releases ω-3 fatty acids such as EPA and DHA from the cell membrane, allowing their metabolism into lipid mediators [63]. *PLA-2* gene expression can be influenced by exposure. For example, *PLA-2* was significantly overexpressed in the lungs of the cigarette smoke- and tobacco smoke-exposed rat lung tissues [64]. The potential involvement of PLA-2 genes in cigarette smoke-induced lung inflammation, exacerbated by tobacco smoke, was further supported by the associated increases in neutrophil infiltration in the BAL fluid and the elevated cytokine and chemokine levels [64]. Previously, AgNP exposure was determined to elevate *iPLA-2* gene expression in both healthy and MetS models, and this elevation was exacerbated in MetS [19]. Our current study confirms the previous discovery, suggesting the unique metabolism of lipid mediators in MetS mice. Our lab previously confirmed that treatment of RvD1 reduced the exacerbation of *iPLA-2* expression in the lung tissue of AgNP-exposed mice [19]. However, RvE1 treatment was determined to not affect *iPLA-2* mRNA expression in healthy mice and resulted in the overexpression of iPLA2 in the MetS model. This suggests that RvE1 treatment did not substantially influence the intracellular availability of fatty acids in either model. Downstream of *PLA-2* are the enzymes responsible for the conversion of ω-3 fatty acids into lipid mediators, which include COX-2 and lipoxygenases (LOX-5/15). Alterations in these enzymes may contribute to the exacerbated inflammatory responses in MetS mice and mediate exposure-induced inflammation [65]. Inhalation exposure to ozone decreases the pulmonary *LOX-5/15* in a mouse model [32]. Similarly, AgNP exposure was determined to decrease *ALOX-5* and *ALOX-15* mRNA gene expression in MetS mice, suggesting alterations in the metabolic processes mediating SPM production [19]. RvE1 promotes the production of anti-inflammatory mediators like lipoxins, which enhance the resolution of inflammation. In allergic inflammation, RvE1 increases lipoxin formation, suggesting an indirect influence on the lipoxygenase pathways [66]. In the current study, RvE1 treatment increased the gene expression of both lipoxygenase enzymes *ALOX-5* and *ALOX-15* in AgNP-exposed MetS mice models. These data imply that RvE1 therapy may restore lipoxygenase activity in MetS animals, allowing the generation of SPMs and reducing inflammatory processes after inhalation exposure.

COX-2 is a well-known enzyme responsible for the conversion of arachidonic acid to the proinflammatory mediator prostaglandin E2 (PGE-2) [67]. The COX-2/PGE-2 pathway is crucial in the development of inflammation induced by PM_2.5_ exposure [68,69]. In our current study, AgNP exposure was determined to elevate *COX-2* mRNA expression in both models. Treatment with RvE1 was previously demonstrated to reduce the expression of NF-kB and *COX-2* Our findings suggest that RvE1 treatment may reduce AgNP-induced inflammation in healthy and MetS models via regulation of *COX-2* production of proinflammatory mediators. Soluble epoxide hydrolase (sEH) is a cytosolic enzyme encoded by the gene *epoxide hydrolase 2* (*Ephx2*) [70,71] that is broadly expressed in bronchi, parenchyma, pulmonary vessels, and macrophages. [71]. sEH hydrolyzes anti-inflammatory epoxy fatty acids, facilitating their clearance [70,72]. Inhibition of sEH may be beneficial for the resolution of inflammation by causing the decreased removal of lipid mediators and allowing increased resolution signaling. In a mouse model, inhibition of sEH prevented bleomycin-induced pulmonary fibrosis [73] and mitigated lipopolysaccharide-induced acute lung injury in mice [74]. Further, a phase I clinical trial recently identified that the sEH inhibitor-GSK2256294 could ameliorate smoking-induced endothelial dysfunction [75], suggesting that the inhibition of sEH has promising therapeutic applications in COPD. In the current study, AgNP exposure elevated *EPHX-2* in both models and RvE1 treatment also increased *EPHX-2* in both models, which would decrease availability of SPMs. This suggests that RvE1 treatment reduces inflammation through mechanisms that appear to be independent of sEH.

RvE1 promotes resolution by binding to the G protein-coupled receptor ChemR-23 [76]. On the cellular level, RvE1 activation of ChemR-23 inhibits TNF-α–induced NF-κB signaling [35] and enhances phagocytosis of apoptotic neutrophils by human macrophages [77]. ChemR-23 is not highly expressed in PMNs, but is present on macrophages [35] where it regulates migration and cytokine production. In our results, *ChemR23* gene expression was elevated in both groups of AgNP-exposed mice, suggesting the engagement of resolution processes at 24 h post-exposure. MetS exacerbated effects may be due to the diminished RvE1 availability. RvE1 treatment reduced ChemR-23 in healthy models compared to the MetS model, thus indicating an ongoing need for resolution in the MetS model.

The metabolism of DHA results in the generation of a variety of SPMs, including PD1 and MaR1, and supplementation of DHA may assist in regulating exposure-induced inflammation [65,78,79]. In Balb/c mouse lungs, dietary DHA therapy reduces inflammatory markers induced by a multi-walled carbon nanotube [80]. Further, pre-treatment with a cocktail of DHA metabolites (14- HDHA, 17-H-DHA, and PDX) reduced ozone-induced lung inflammation in a healthy mouse model [32]. These studies demonstrate that treatments to increase DHA-derived SPMs can reduce pulmonary inflammation following exposure. It is likely that diseases and exposure result in dysregulation of specific SPMs, contributing to inflammatory responses. Therefore, to support the development of treatments, it is necessary to understand their distinct abilities to regulate inflammation. For example, we previously determined that treatment with DHA-derived RvD1 benefited MetS mice by reducing the inflammatory response to levels observed in healthy mice following AgNP-exposure, without altering responses in the healthy model [19]. To further understand the distinct roles of other DHA-derived SPMsm we treated mice with either PD1 or maresin-1.

PD1 treatment has demonstrated significant attenuation of histological damage, neutrophil infiltration, and proinflammatory cytokine generation following LPS-induced acute lung injury in mice [41]. In humans, PD1 levels were determined to be lower in obese patients compared to healthy controls, suggesting that diminished PD1 could contribute to sustained inflammation in obese people [81]. Our current study reinforces these findings and suggests PD1 as a potential treatment to reduce the aggravated pulmonary inflammation observed in the MetS model following AgNP exposure. Specifically, PD1 treatment reduced the exacerbation of AgNP-induced pulmonary neutrophilia in the MetS model, without affecting the neutrophil counts in AgNP-exposed healthy mice. PD1 treatment decreased the levels of *CCL-2*, *IL-6*, and *CXCL-1* mRNA expression in both models exposed to AgNPs. While the PD1 treatment did not completely reduce the protein level of IL-6, it did reduce the exacerbated amount of IL-6 protein level in the MetS model exposed to AgNPs. The sustained presence of IL-6 protein may be responsible for the presence of neutrophils observed following PD1 treatment in the AgNP-exposed MetS model. Additionally, PD1 treatment influenced IL-10 production, which may also contribute to changes in AgNP-induced inflammation. Previously, treatment with protectin DX (PDX), an isomer of PD1, increased the proportion of M2 macrophages in septic mice and elevated levels of IL-10 were also observed [82,83]. Additionally, treatment of monocyte-derived macrophages with n–3 PUFA-rich lipid emulsion, which induces PDX release, significantly increased the IL-10 secretion [84]. Our results support these previous findings demonstrating that PD1 treatment elevates IL-10 production, which may facilitate resolution.

PD1 is an ω-3 DHA-derived lipid mediator generated via a coordinated metabolism process involving multiple enzymes. This process begins with iPLA-*2* cleavage of DHA from the cell membrane. In our current study, the over-expression of the *iPLA-2* gene was observed in both AgNP-exposed models and was exacerbated in MetS mice, leading to potentially increased intracellular DHA from metabolism into the lipid mediators. PD1 treatment completely reduces the *iPLA-2* in both the healthy and MetS AgNP-exposed models. This suggests that PD1 treatment may reduce AgNP-induced inflammation in healthy and MetS models via the regulation of iPLA-2. Previously, exposure to DHA-derived RvD1 was determined to significantly attenuate *COX-2* expression [85]. Our current study determined that PD1 treatment significantly reduces *COX-2* expression in the lung tissue of AgNP-exposed mice, suggesting the potent anti-inflammatory function of PD1. PD1 signals via *GPR-37* activation of macrophages resulting in increased phagocytosis, altered cytokine release, and the promotion of resolution. Knock-out of *GPR-37* impairs the in vivo macrophage phagocytosis of zymosan and apoptotic neutrophils, but has no effect on neutrophil recruitment or on the reduction in neutrophil numbers at later time points. In the current study, *GPR-37* was elevated in both AgNP-exposed models and was enhanced in the MetS model, while PD1 treatment reduced the *GPR-37* in both models. This downregulation of *GPR-37* suggests negative feedback due to the activation of GPR37 by the PD1provided. Based on previous evaluations of PD1 signaling via GPR-37, it is likely that PD1 may benefit mice exposed to AgNPs at later time points by the stimulation of efferocytosis.

Maresin-1 (MaR1) is a macrophage-derived pro-resolving lipid mediator also synthesized from DHA [86,87]. MaR1 suppresses the activation of the NLRP3 inflammasome, the Th2-type immunological response, and oxidative stress [88,89]. Further, MaR1 significantly lowered BALF neutrophil infiltration and intracellular adhesion molecule-1 (ICAM-1) expression in mice with organic dust-induced airway inflammation [45]. Our current study supports previous findings in that MaR1 treatment reduced AgNP exposure-induced neutrophil infiltration in both healthy and MetS models. Previous evaluations have demonstrated that MaR1 accelerates resolution by reducing the production of proinflammatory cytokines. For example, MaR1 treatment in a LPS-induced acute lung injury model demonstrated reduced production of pulmonary proinflammatory cytokines [43]. MaR1 treatment also reduced cytokine production, including *IL-6*, *IL-8*, and *TNF-α*, in HDE-exposed bronchial epithelial cells [90]. Our current study demonstrated that MaR1 treatment inhibits the production of proinflammatory cytokines *IL-6* and *CXCL1* in response to AgNP exposure in both healthy and MetS models. Although some cytokines remained upregulated in the MetS model exposed to AgNPs and treated with MaR1, this may have been counteracted by the substantial upregulation of *IL-10*. Other studies have demonstrated that MaR1 treatment upregulates IL-10, suggesting that IL-10 mediates some of MaR1’s resolution capacity [91,92,93].

MaR1 treatment may also influence lipid metabolism processes, affecting their signaling. In the current study, MaR1 treatment completely inhibited the AgNP-induced expression of *iPLA-2* in both models, suggestive of alterations in the available precursors of lipid mediators. MaR1 is synthesized from DHA by enzymes such as 12-lipoxygenase (12-LOX), 12/15-LOX, and 5-LOX [94]. Our current study shows that MaR1 treatment increases *ALOX-5* levels in both AgNP-exposed models; however, it particularly upregulates *ALOX-15* in MetS models compared to healthy models. This finding suggests that MaR1 treatment may stimulate the resolution of inflammation through modulation of *ALOX-15* in MetS mice. MaR1 treatment suppresses the activation of the NF-κB signaling pathway, reducing COX-2 and ICAM-1, preventing inflammatory cell infiltration in the BALF and excessive mucus production [95]. Treatment with MaR1 lowers *COX-2* expression, which reduces the generation of prostaglandin E2, an essential mast cell mediator and potent immunomodulator that promotes inflammation in asthmatic human airway epithelial tissues [96]. Similarly, in our study, MaR1 treatment reduced *COX-2* expression in both healthy and MetS mice models, which may have resulted in the diminishment of inflammation. Lastly, we determined that MaR1 treatment decreases *EPHX-2* expression in the lung tissue of both healthy and MetS models exposed to AgNPs. This suggests that lipid mediators may not be removed as efficiently, leading to enhanced signaling via their receptors. MaR1 stimulates resolution through *LGR-6* receptor activation [97]. LGR-6 receptor activation by MaR1 inhibits the pulmonary artery’s smooth muscle cell proliferation in vitro through decreased phosphorylation of STAT3, AKT, ERK, and FoxO1 [98]. Our study found that exposure to AgNPs increased *LGR-6* expression in healthy mice but had no effect on MetS mice, suggesting a decreased capacity for MaR1 to stimulate resolution in MetS mice. Treatment with MaR1 increased *LGR-6* expression in healthy mice, particularly in the MetS model. This suggests that MaR1 potentially activates LGR-6 in AgNP-exposed mice to promote the resolution of lung inflammation.

Our evaluation was crucial for detecting changes in pulmonary inflammatory markers due to underlying health conditions and identifying the specific deficiencies in resolution signaling that contribute to susceptibility to inhalation exposure in MetS models. However, it is important to acknowledge certain limitations of our evaluation. Producing a human equivalent to the MetS mouse model is challenging due to the differences in cholesterol metabolism. Specifically, mice lack the cholesterol ester transfer protein (CETP) [99] found in humans, resulting in higher HDL and lower LDL and triglyceride levels than in humans on high-fat diets. However, our selected mice model is widely accepted for evaluating MetS because it accurately replicates the major disease components [100,101,102]. Additionally, we utilized a relatively high concentration of AgNPs to induce inflammation. This concentration was selected to replicate previous research and provoke an inflammatory response to evaluate changes caused by both disease and treatment. To gain a more comprehensive understanding of susceptibility and inflammatory signaling, future studies would benefit from employing lower concentrations and/or repeated exposures. Investigations of sustained inflammation examining multiple time points and long-term impacts are necessary to understand SPM-mediated signaling deficiencies in MetS.

## 5. Conclusions

Research continues to link environmental inhaled particulates with dysregulated synthesis of proinflammatory and pro-resolving lipid mediators in the lungs. MetS is linked to increased susceptibility to inflammation and toxicity caused by inhaling particulate matter. Treatments addressing lipid mediator dysregulation may be impactful as therapeutics for exposure-induced inflammation. In our study, RvE1 treatment completely reduced the neutrophilia induced by AgNPs in healthy mice, and only the exacerbated neutrophilia presented in the exposed MetS model. PD1 treatment did not affect the neutrophil cell count in AgNP-exposed healthy mice, however, it significantly reduced the exacerbated neutrophilia in the AgNP-exposed MetS model. Lastly, MaR1 treatment completely reduced AgNP-induced neutrophilia in both models. These modifications in inflammatory responses are likely due to the different signaling capacities of SPMs in terms of resolution. For example, all of the selected treatments are known to inhibit proinflammatory cytokine production. However, RvE1 promotes efferocytosis and inhibits dendritic cell migration, while PD1 enhances IL-10 production and inhibits dendritic cell migration. MaR1 functions by the promotion of efferocytosis, stimulating IL-10 production, shifting macrophages towards M2-like phenotypes, and inhibiting major inflammatory intracellular signaling pathways (STAT1, STAT3, STAT5, p38, and ERK1/2). The patterns of response to RvE1, PD1, and MaR1 treatment suggest that differential mechanisms are altered following exposure in healthy and MetS mice, contributing to the differences in susceptibility. Overall, the use of SPMs (specifically RvE1, PD1, and MaR1) appeared to distinctly alleviate AgNP-induced inflammation, suggesting that SPM therapies might reduce the lung inflammation caused by inhaling particulate matter in certain susceptible subpopulations.

## Data Availability

Data is contained within the article.
